# PASSING THE TORCH: EXPLORING THE SUSTAINABILITY OF AN INTERPROFESSIONAL GERIATRICS FACULTY DEVELOPMENT PROGRAM

**DOI:** 10.1093/geroni/igac059.404

**Published:** 2022-12-20

**Authors:** Kimberly Davis, Sarah Marrs, Ishan Williams, Kristin Zimmerman, Constance Coogle, Pamela Parsons, Patricia Slattum, Leland Waters

**Affiliations:** Virginia Commonwealth University/ School of Nursing, Richmond, Virginia, United States; Virginia Commonwealth University, Richmond, Virginia, United States; University of Virginia, School of Nursing, Charlottesville, Virginia, United States; Virginia Commonwealth University, School of Pharmacy, Richmond, Virginia, United States; Virginia Commonwealth University, Richmond, Virginia, United States; Virginia Commonwealth University, Richmond, Virginia, United States; Virginia Commonwealth University, Richmond, Virginia, United States; Virginia Commonwealth University, Richmond, Virginia, United States

## Abstract

Faculty development programs (FDPs) are an effective, evidence-based method of promoting the knowledge, skills and self-efficacy of faculty. However, the implementation and sustainability of curricula are rarely reported. Furthermore, the challenges to sustaining programmatic implementation of interprofessional FDP curricula in academic and clinical settings over time have yet to be extensively evaluated. Using dynamic sustainability as a framework, we aimed to assess the evolving barriers and facilitators that influence the implementation and sustainability of the geriatrics curriculum Capstone projects designed by faculty scholars in our FDP. We planned to report on projects that were and weren't successful. A survey, sent to 115 faculty scholars from eight different cohorts who completed our 10-month FDP, recruited faculty participants and set the stage for qualitative data collection to help us better understand the sustained impact of the program. Thematic analysis of virtual interviews with 17 Scholars revealed several key factors facilitating and hindering the implementation and dynamic sustainability of curricular projects. Three major themes and sub-themes were identified as follows: Project Implementation (Supportive Factors, Hindering Factors and Filling in Gaps in the Field); Pedagogical Development (Enhancement of Skills and Culture Change); and Sustainability Impact (Project Sustainability, Career Development and Passing the Torch). Supporting these factors through skills development may favorably impact project sustainability and thus the aspects of workforce development that catalyze practice change. Implementation of geriatrics-focused FDPs provides an evidence-based approach to sustainability. Further study of the ongoing barriers and facilitators to sustainability is encouraged.

